# The Emerging Role of Ubiquitin-Specific Protease 36 (USP36) in Cancer and Beyond

**DOI:** 10.3390/biom14050572

**Published:** 2024-05-12

**Authors:** Meng-Yao Niu, Yan-Jun Liu, Jin-Jin Shi, Ru-Yi Chen, Shun Zhang, Chang-Yun Li, Jia-Feng Cao, Guan-Jun Yang, Jiong Chen

**Affiliations:** 1 State Key Laboratory for Managing Biotic and Chemical Threats to the Quality and Safety of Agro-Products, Ningbo University, Ningbo 315211, China; 216004832@nbu.edu.cn (M.-Y.N.); 2201130072@nbu.edu.cn (Y.-J.L.); 2311130039@nbu.edu.cn (J.-J.S.); 2311130002@nbu.edu.cn (R.-Y.C.); 2211130023@nbu.edu.cn (C.-Y.L.); caojiafeng@nbu.edu.cn (J.-F.C.); 2 Laboratory of Biochemistry and Molecular Biology, School of Marine Sciences, Meishan Campus, Ningbo University, Ningbo 315832, China; 3 Ningbo No.2 Hospital, Laboratory of Biochemistry and Molecular Biology, School of Marine Sciences, Meishan Campus, Ningbo University, Ningbo 315832, China; zhangshun@ucas.ac.cn

**Keywords:** USP36, cancer, ubiquitination, deubiquitinating enzyme

## Abstract

The balance between ubiquitination and deubiquitination is instrumental in the regulation of protein stability and maintenance of cellular homeostasis. The deubiquitinating enzyme, ubiquitin-specific protease 36 (USP36), a member of the USP family, plays a crucial role in this dynamic equilibrium by hydrolyzing and removing ubiquitin chains from target proteins and facilitating their proteasome-dependent degradation. The multifaceted functions of USP36 have been implicated in various disease processes, including cancer, infections, and inflammation, via the modulation of numerous cellular events, including gene transcription regulation, cell cycle regulation, immune responses, signal transduction, tumor growth, and inflammatory processes. The objective of this review is to provide a comprehensive summary of the current state of research on the roles of USP36 in different pathological conditions. By synthesizing the findings from previous studies, we have aimed to increase our understanding of the mechanisms underlying these diseases and identify potential therapeutic targets for their treatment.

## 1. Introduction

Deubiquitinating enzymes (DUBs), also known as deubiquitinases, consist of a specific class of proteases that exert their functions via removing ubiquitin tags from substrate proteins. DUBs are associated with physiological and pathological processes that modulate various cellular processes. The repertoire of human DUBs encompasses approximately 100 enzymes that are categorized into seven distinct families: ubiquitin-specific proteases (USP/UBP), ubiquitin C-terminal hydrolases (UCHs), ovarian tumor proteases (OTUs), JAB1/MPN/MOV34s (JAMMs), josephins, zinc finger proteins containing UFM1-specific peptidase domains such as ZUFSP, and the recently characterized MINDY protease [1,2,3]. The USP family, composed of more than 50 members, represents the most extensive and heterogeneous subset within the DUB family [4,5]. These USPs demonstrate selectivity towards ubiquitin and play a critical role in governing protein turnover through ubiquitination, a pivotal post-translational modification.

USP36 is a member of the USP family that contributes to cellular homeostasis and disease progression by preventing the degradation of diverse target proteins involved in tumorigenesis and inflammatory responses via removing K48-linked ubiquitin chains. Notably, USP36 stabilizes nucleolar activity via deubiquitinating nucleophosmin/B23 and fibrillarin [6,7]. This enzyme has also been linked to autophagy activation [8] and the regulation of oxidative stress by stabilizing mitochondrial superoxide dismutase SOD2 via reducing its ubiquitination [9]. USP36 has been implicated in regulating ribosome biogenesis through its interaction with RNA polymerase I [10,11].

Increasing interest in exploiting USP36 as a therapeutic target has prompted the extensive exploration of its protein interactions and functional significance. This review underscores the importance of comprehending the protein interactions of USP36, particularly in the context of their implications for cancer and other biological processes. These investigations have not only provided insights into the role of USP36 in tumorigenesis but have also revealed its involvement in diverse cellular pathways, including inflammatory responses, autophagy activation, oxidative stress regulation, and ribosome biogenesis. By unraveling the intricacies of USP36 protein interactions and its functional roles, researchers are gaining a more thorough understanding of its molecular underpinnings and also its potential as a target for therapeutic interventions, transcending its relevance solely in the realm of cancer research. Such endeavors hold promise for the development of novel treatments that leverage the multifaceted functions of USP36.

## 2. The Structure of USP36

**Figure 1:**
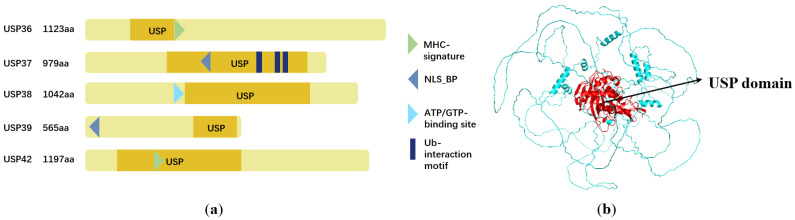
The structure of USP36. (**a**) Comparison of the domain structures of putative USPs. (**b**) The structure of USP36 is predicated by AlphaFold, and its USP domains (amino acid residues 122–423) are labeled in red.

The human USP36 gene is located on chromosome 17q25.3, and the USP36 mRNA encodes an 1123 amino acid polypeptide. The molecular weight of the residual polypeptide is approximately 122.907 kilodalton (kDa) and the isoelectric point is predicted to be 9.73 based on the Expasy server (https://www.expasy.org (accessed on 20 December 2023)). Within the human USP subfamily, comprising a total of 56 members, USP36 and USP42 belong to the same subcluster based on phylogenetic classification. This classification is supported by the significant structural similarity between the two proteins, highlighting that their amino acid sequences share a considerable degree of identity, reaching 62%, and have a conserved USP catalytic domain (Figure 1a,b).

## 3. Biological Function of USP36

### 3.1. Classical Deubiquitination Activity of USP36

Ubiquitination, a prevalent form of protein post-translational modification, plays a crucial role in controlling various cellular functions [12]. This reversible process can be catalyzed by a series of deubiquitinating enzymes known as deubiquitinases (DUBs) [13,14]. DUBs may enhance protein stability and modify signaling pathways to modulate the function of specific proteins, significantly influencing a range of malignancies. These deubiquitinating enzymes are part of the ubiquitin-specific protease (USP) family. Recent research indicates that USP36 serves as a key regulator, modulating numerous cellular biological processes through deubiquitination. This process consequently reduces the proteasomal degradation of proteins implicated in tumorigenesis.

### 3.2. The Functions of USP36 in Nucleolar Protein SUMOylation

SUMOylation is a pivotal regulatory mechanism in various cellular processes including ribosomal biogenesis. Proteomic investigations and empirical data have revealed that numerous nucleolar proteins implicated in ribosome biogenesis undergo SUMO modifications [15]. Proteins associated with ribosome biogenesis form a prominent subset of functionally interlinked targets for SUMOylation within cells [16], underscoring the pivotal role of SUMOylation in nucleolar ribosome biogenesis [17,18,19]. USP36 can facilitate nucleolar SUMOylation both in vitro and in vivo through direct interaction with SUMO2 and Ubc9. Research indicates that USP36 augments the SUMOylation of small nucleolar ribonucleoprotein (snoRNP) components Nop58 and Nhp2, thereby enhancing their binding affinity to snoRNAs. Furthermore, USP36 also promotes the SUMOylation of snoRNP components Nop56 and DKC1. Functional studies have demonstrated that the knockdown of USP36 significantly impacts rRNA processing and translation. Consequently, USP36 plays a crucial role in ribosome biogenesis and protein translation by facilitating the SUMOylation of snoRNP components [15].

USP36 engages in a mutual interaction with EXOSC10 under hypoxic conditions, thereby facilitating the SUMOylation of EXOSC10. Furthermore, USP36 can also partake in the SUMOylation of EXOSC10 in conjunction with NPM1. However, this interaction between USP36 and EXOSC10 is disrupted under hypoxic conditions, leading to the loss of SUMOylation of EXOSC10 [20]. USP36 facilitates the SUMOylation of EXOSC10 by functioning as a SUMO E3 enzyme, thereby enhancing nucleolar RNA exoribonuclease functionality and cell proliferation. While USP36 directly interacts with EXOSC10, it does not markedly influence the levels of EXOSC10 or other examined exoribonuclease subunits. Instead, it orchestrates the SUMOylation of EXOSC10 at Lysine (K)583. A mutation in K583 impedes the binding of EXOSC10 to pre-rRNAs, and the K583R mutant fails to rectify rRNA processing deficiencies and cell growth inhibition induced by endogenous knockdown of EXOSC10. Additionally, when ribosome biogenesis is disrupted, the SUMOylation of EXOSC10 is significantly diminished. Consequently, USP36 is pivotal in nucleolar RNA exoribonuclease function by enabling EXOSC10 SUMOylation through its role as a SUMO ligase [21].

USP36 modulates the biogenesis of miRNA through the SUMOylation of DGCR8. This interaction occurs between USP36 and the microprocessor complex, promoting the SUMOylation of DGCR8, with a particular emphasis on SUMO2-modified DGCR8. The role of USP36 in this process does not influence the concentration of DGCR8 or the formation of the Drosha-DGCR8 complex. However, it enhances the binding of DGCR8 to pri-miRNAs. Consequently, USP36 controls the processing of pri-miRNA within the nucleolus via the SUMOylation of DGCR8 [22].

## 4. USP36-Mediated Signaling Pathway

**Figure 2:**
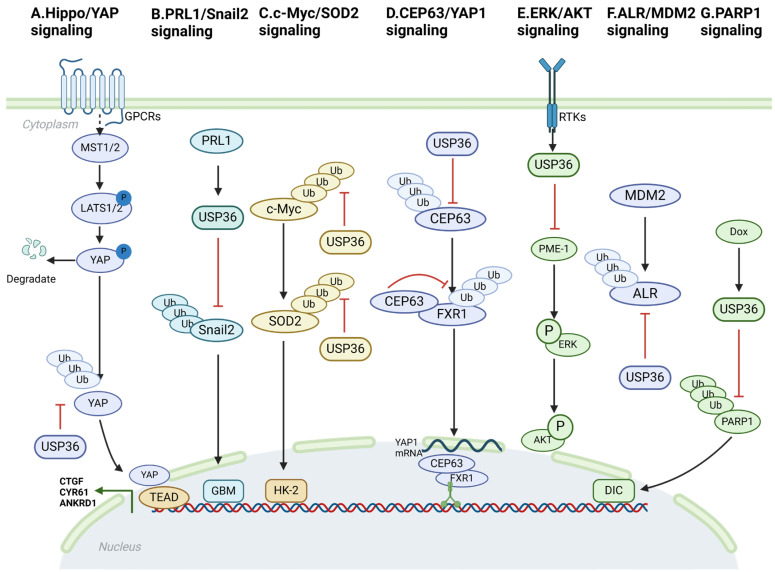
The overview of USP36-mediated signaling pathways. (**A**) Hippo/YAP signaling. USP36 stabilizes the YAP protein by inhibiting the polyubiquitination of the K48 chain of the YAP protein. First, USP36 binds to the YAP protein to form a stable complex. Second, USP36 prevents the degradation of YAP protein by inhibiting the polyubiquitination of the K48 chain of YAP protein. Finally, stable YAP protein can continue to participate in the regulation of Hippo/YAP signaling pathway, thereby affecting the growth and differentiation of cells. (**B**) PRL1/Snail2 signaling. In glioblastoma (GBM), PRL1 promotes tumor invasion and metastasis by activating USP36-mediated deubiquitination of Snail2. (**C**) c-Myc/SOD2 signaling. Overexpression of USP36 is accompanied by increased levels of c-Myc and SOD2 proteins, attenuating ischemia-induced ubiquitination of these two proteins. (**D**) CEP63/YAP1 signaling. USP36 can promote YAP1 expression by binding to CEP63 and inhibiting K63 ubiquitination degradation of FXR1, thereby enhancing cancer stem cell properties. (**E**) ERK/AKT signaling. USP36 regulates the stability of PM-1. Through deubiquitination, USP36 decreases the expression level of PME-1 and promotes the activation of ERK and Akt signaling pathways. (**F**) ALR/MDM2 signaling. MDM2 promotes the degradation of ALR proteins by ubiquitinating them, while USP36 maintains the stability of ALR proteins by removing ubiquitination modifications. (**G**) PARP1 signaling. Dox promotes the progression of DIC by activating USP36-mediated deubiquitination of PARP1. This figure was produced by Biorender (Agreement number: YM26R6CFJU).

USP36 modulates the activity of the Hippo/YAP, PRL1/Snail2, c-Myc/SOD2, CEP63/YAP1, Fbw7g/USP36/c-Myc, ERK/AKT ALR (augmentation of liver regeneration)/MDM2, and USP36/PARP1 pathways through interaction with specific components and exertion of deubiquitinating effects (Figure 2). These interactions enable USP36 to affect the activation, stability, and downstream gene expression of pivotal proteins within these pathways. The dysregulation of these signaling cascades has been well established as a factor contributing to the initiation, promotion, and also progression of cancer.

### 4.1. Hippo/YAP Signaling

The Hippo signaling pathway was initially identified in Drosophila melanogaster as a mechanism governing organ size control [23]. Subsequent investigations have identified similar functions in humans. This evolutionarily conserved pathway plays a critical role in orchestrating tissue regeneration, maintaining stem cell populations, and influencing cancer development. It functions through a complex network of protein interactions, phosphorylation events, and transcriptional modulation [23]. The fundamental constituents of the Hippo pathway are centered on a phosphorylation cascade. The upstream Hippo kinase MST1/2 initiates this cascade by phosphorylating downstream LATS1/2 kinases. Consequently, phospho-LATS1/2 kinases are involved in the phosphorylation of YAP at multiple serine residues. This phosphorylation enables the sequestration of YAP within the cytosol, ultimately leading to proteasome-dependent degradation [24,25]. YAP, a pivotal downstream mediator of the Hippo signaling pathway, acts as a cofactor for a diverse variety of transcription factors, notably TEADs [26]. Furthermore, the YAP protein has the capacity to activate additional signaling pathways beyond its role as a coactivator for transcription factors, including the Hedgehog and Wnt signaling pathways [27,28]. Numerous previous studies have demonstrated that the upregulation of YAP protein expression in human malignancies is correlated with advanced tumor stage and unfavorable prognostic outcomes [29]. Therefore, an effective therapeutic strategy for human cancers could involve blocking or inhibiting YAP, which could activate the tumor-suppressive function of Hippo signaling. Activating receptors for Hippo/YAP signaling include TAZ, YAP, and LPA1-6 [30]. The findings of the current study highlight the significance of USP36 in the modulation of Hippo signaling activity and growth of esophageal squamous cell carcinoma (ESCC). It stabilizes YAP proteins by inhibiting the polyubiquitination of the K48 chain of YAP proteins (Figure 2A). Thus, targeting Hippo/YAP signaling may be a viable therapeutic approach for patients with ESCC [31].

Recent studies have revealed a previously unknown biological association between the Hippo–YAP axis and USP36 expression in ESCC. These findings indicate that USP36 interacts with YAP and plays a role in deubiquitinating YAP, thereby positively influencing Hippo/YAP signaling activity. These results provided valuable insights into the regulatory mechanisms of the Hippo pathway in ESCC. Considering the significance of this discovery, targeting the expression or function of USP36 may hold promise for the treatment of ESCC. By modulating USP36 levels or activity, it may be possible to influence Hippo/YAP signaling and potentially impede the progression of ESCC. However, further research is imperative in order to fully comprehend the complex molecular mechanisms underlying this interaction and to determine the feasibility and effectiveness of targeting USP36 as a therapeutic strategy for ESCC [31].

### 4.2. PRL1/Snail2 Signaling

Phosphatases of regenerating liver (PRLs) belong to a subgroup within the protein tyrosine phosphatase (PTP) family comprising PRL1, PRL2, and PRL3. These phosphatases are involved in development and the metastatic process [32]. PRL1, which is encoded by the PTP4A1 gene in humans, was originally discovered as an upregulated marker of liver regeneration following partial hepatectomy [33]. Cancer cells are influenced by the Snail family members Snail1 and Snail2 [34]. Snail proteins undergo polyubiquitination and subsequent degradation mediated by the 26S proteasome, a process that is regulated by their phosphorylation status [35,36]. PRL1 is a well-established oncogene involved in several types of cancers. This study demonstrated the substantial upregulation of PRL1 in glioma tissues and cell lines, which exhibited a positive correlation with tumor grade. Moreover, elevated levels of PRL1 protein are positively associated with the expression of Snail2 and indicate an unfavorable prognosis in cancer cells [37]. In this study, we explored the potential role of PRL1 in tumorigenesis and invasion. These findings revealed that PRL1 is upregulated in cancer cells and is correlated with patient prognosis. Ectopic expression and knockdown experiments were conducted in order to investigate the functional significance of these genes. These results demonstrated that increased levels of PRL1 promote invasion and tumorigenesis, indicating an oncogenic role for PRL1. Conversely, PRL1 knockdown had the opposite effect, inhibiting invasion and tumorigenesis. These observations suggest that PRL1 plays a significant role in tumor progression and invasion, making it a potential target for therapeutic interventions aimed at mitigating cancer metastasis. Further investigation found that PRL1 plays a crucial role in the deubiquitination and stabilization of Snail2. This process is facilitated by the activation of USP36. These findings suggest that targeting the PRL1/USP36/Snail2 axis could potentially inhibit epithelial–mesenchymal transition (EMT) and reduce tumor invasion and metastasis, making it an attractive target for future therapeutic interventions (Figure 2B).

### 4.3. c-Myc/SOD2 and c-Myc/Fbw7g Signaling

Ischemic conditions significantly contribute to acute kidney injury (AKI), with a particular emphasis on ischemia-induced kidney injury. Ischemia induces mitochondrial respiratory disturbances and escalates electron leakage within the electron transport chain (ETC). These alterations may result in a reduction of SOD2 expression or activity, thereby influencing antioxidant stress. Oncoprotein c-Myc, also known as MYC, plays a regulatory role in a wide range of cellular processes. These processes include cell-cycle progression, nucleotide biosynthesis, metabolism, RNA processing, ribosomal biogenesis, protein translation, apoptosis, senescence, and differentiation [38]. MYC functions as a pleiotropic transcription factor in conjunction with its primary partner, MAX [39]. This partnership is facilitated by the basic C-terminal HLH/Leu-zipper domain (bHLH-LZ) [40]. Through the formation of MYC/MAX heterodimers, numerous target genes are activated by binding to canonical E-box (CACGTG) elements in gene promoters and enhancers [41]. Furthermore, ischemia triggers an increase in c-Myc protein levels, intensifying oxidative stress. The researchers discovered that USP36 functions as a unique deubiquitinating enzyme capable of interacting with and deubiquitinating c-Myc. This interaction inhibits c-Myc degradation and enhances c-Myc signaling. The implications of these findings may play a significant role in the pathogenesis of AKI. Furthermore, it was discovered that USP36 has the capacity to deubiquitinate SOD2 within mitochondria, which subsequently impacts cellular respiration. These findings imply that USP36 could potentially influence oxidative stress and the protective functions of kidney cells by modulating the ubiquitination of c-Myc and SOD2. Consequently, comprehending the regulation of c-Myc and SOD2 via ubiquitination holds significant importance for investigating the pathogenesis of AKI and identifying novel intervention targets (Figure 2C) [42].

SCF-Fbw7 is the most extensively studied ubiquitin E3 ligase involved in MYC regulation. The core constituents of the SCF complex consist of an adaptor protein known as Skp1, scaffold protein Cul1, and ring-finger protein Rbx1/Roc1. Fbw7 serves as an F-box protein within this complex and functions as a variable element that confers its substrate specificity [43]. There exist three distinct isoforms of Fbw7, which exhibit differential subcellular localization: Fbw7α predominantly localizes to the nucleoplasm, Fbw7β is primarily found in the cytoplasm, and Fbw7γ is specifically localized within the nucleolus [43,44]. Both Fbw7α and Fbw7γ isoforms have been demonstrated to facilitate the degradation of MYC. The turnover of MYC by Fbw7 is contingent on a sequential phosphorylation event at two specific sites: S62 and T58. Upon receiving growth signals, MYC stability is enhanced through S62 phosphorylation, which is mediated by the Ras-Raf-MEK-ERK kinase cascade and/or cyclin-dependent kinases (CDKs) [45,46,47]. USP36 plays a direct role in MYC stabilization by interacting with and deubiquitinating MYC. Interestingly, USP36 interacted specifically with the nucleolar Fbw7g isoform but not with the nucleoplasmic Fbw7a isoform. Despite this, USP36 could effectively inhibit MYC degradation initiated by both Fbw7g and Fbw7α, thereby indicating that USP36 governs the final stage of Fbw7-mediated MYC degradation within the nucleolus, while cooperating with USP28 in the nucleoplasm. This is further supported by the observation that USP36 and USP28 exhibit a synergistic effect on increasing MYC levels [48]. Based on the available information, it is plausible that the Fbw7a-USP28 and Fbw7g-USP36 axis is responsible for dynamically regulating the life cycle of MYC in distinct cellular compartments. USP36 has been identified as a target gene of MYC, implying the presence of a positive feedback regulatory loop between USP36 and MYC. This suggests that MYC can enhance its stability by upregulating the expression of USP36, further reinforcing the regulatory control over MYC levels [49]. The observation of elevated USP36 expression in certain types of human breast and lung cancers suggests is involvement in promoting oncogenesis. Additionally, the identification of MYC as a target of USP36 further supports the crucial role of USP36 in ribosome biogenesis. This indicates that USP36 plays a critical role in the cellular processes related to the production of ribosomes, which are essential for protein synthesis and cell growth. Dysregulation of USP36 and its interaction with MYC may contribute to aberrant ribosome biogenesis and potentially drive cancer development [6,50,51].

### 4.4. CEP63/YAP1 Signaling

CEP63, a constituent of the centriole, has been found to exhibit upregulation in neuroblastoma. This upregulation has been associated with unfavorable prognostic outcomes in individuals with this type of cancer. Overexpression of CEP63 highlights its potential role as a molecular marker for disease progression and prognostic assessment in patients [52]. Previous research has provided evidence that CEP63 exerts regulatory control over centriole biogenesis and asymmetric distribution through its involvement in protein complexes, namely the WDR62-ASPM-CEP63 complex or the CEP63-CEP152 complex. These intricate molecular interactions are crucial for maintaining the characteristic properties of stem cells, highlighting the significance of CEP63 in the cellular development and preservation of stemness [53,54]. These studies revealed that CEP63 can deubiquitinate and stabilize the RNA-binding protein, FXR1. This interaction facilitates the regulatory influence of FXR1 on YAP1 mRNA expression, thereby promoting the acquisition and maintenance of stem cell phenotypes in colorectal cancer (CRC). Notably, this effect was independent of the traditional centrosome-related functions of CEP63. Hence, akin to numerous other centrosome constituents, CEP63 has been shown to exhibit multifaceted roles in cellular processes beyond its canonical involvement in centrosome function. USP36 functions as a deubiquitinating enzyme, which stabilizes the CEP63 protein and consequently augments its K48-dependent deubiquitination. The interaction between CEP63 and FXR1 is not reliant on RNA. Furthermore, microtubule motor proteins have the capacity to form complexes with both CEP63 and FXR1, thereby influencing the regulation of RNA targets by FXR1 (Figure 2D) [55].

### 4.5. ERK/AKT Signaling

Protein phosphatase 2A (PP2A) functions as a tumor suppressor, and its inactivation is associated with the development of solid tumors and hematological malignancies [56,57]. Inactivation of PP2A can be attributed to the action of protein phosphatase methylesterase 1 (PME-1), leading to the suppression of the ERK and AKT signaling pathways. These signaling pathways play crucial roles in cellular processes such as cell proliferation and survival. They also serve as essential mediators of oncogenic pathway activation. Mutations in the genes encoding components of the ERK and AKT signaling pathways have been associated with an elevated incidence of cancer. Previous studies demonstrated that USP36 can increase the expression of PME-1 by facilitating K48-linked polyubiquitination. Conversely, suppression of USP36 activity leads to a reduction in expression levels of PME-1 [58]. In summary, these findings indicate that USP36 functions as a deubiquitinase to modulate PME-1 and promote the ERK and AKT signaling pathways (Figure 2E). Thus, targeting USP36 may represent a potential therapeutic approach for attenuating the proliferation and survival of cancer cells.

### 4.6. ALR/MDM2 Signaling

ALR functions as a sulfhydryl oxidase and cytochrome c reductase, playing a key role in the regulation of mitochondrial dynamics [59]. The interaction mechanism between ALR and USP36 involves the regulation of ALR protein stability. This is achieved through the interplay of the E3 ubiquitin ligase, MDM2, and the deubiquitinating enzyme, USP36. Specifically, MDM2 facilitates the degradation of ALR proteins by inducing ubiquitination, whereas USP36 counteracts this degradation by eliminating ubiquitination modifications [59] (Figure 2F).

### 4.7. USP36/PARP1 Signaling

Anthracyclines, notably doxorubicin (Dox), are extensively utilized as chemotherapeutic agents with broad-spectrum efficacy against various hematologic malignancies and solid organ tumors [60,61]. Nonetheless, akin to its anthracycline counterparts, the principal clinical drawback of Dox is the occurrence of cardiotoxicity, distinguished by cardiomyocyte apoptosis, fibrosis, hypertrophy, and cellular vacuolization [62]. The reduction in USP36 expression demonstrated a significant attenuation of Dox-induced cardiotoxic effects both in vitro and in vivo. Mechanistically, USP36 physically interacts with poly (ADP-ribose) polymerase 1 (PARP1) to regulate PARP1 stability by inhibiting its ubiquitination. Additionally, PARP1 mediates the effect of USP36 on Dox-induced cardiotoxicity modulation. This investigation provides compelling evidence that USP36 deubiquitinates PARP1, suggesting that the USP36/PARP1 axis is a promising therapeutic target for Dox-induced cardiomyopathy [63] (Figure 2G).

## 5. Role of USP36 in Diseases

**Figure 3:**
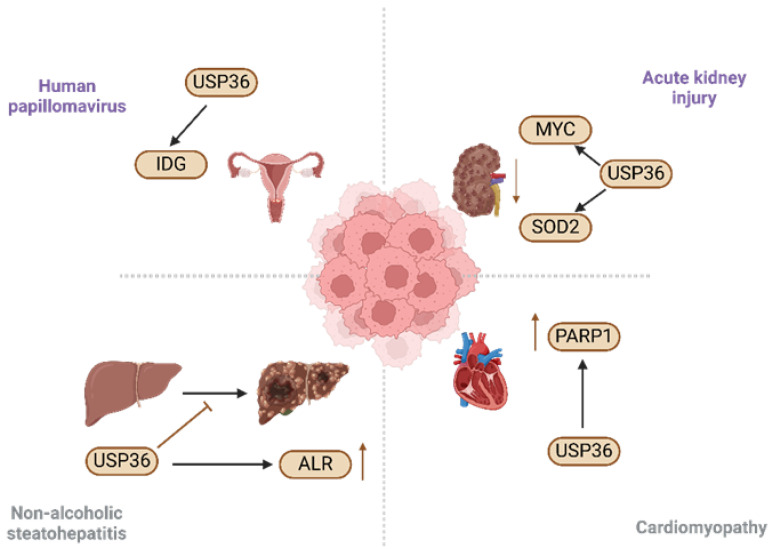
The role of USP36 in non-cancerous diseases. This figure was produced by Biorender (Agreement number: JM26L98JPQ).

Recent studies have revealed a significant correlation between USP36 expression and various human diseases. USP36 has been implicated in a range of non-cancerous conditions, including AKI [42], nonalcoholic steatohepatitis [59], human papillomavirus infection [64], and cardiomyopathy [63]. Hence, elucidating the association between USP36 and these diseases, along with the underlying regulatory mechanisms, is important for disease prevention and therapeutic intervention (Figure 3).

### 5.1. Acute Kidney Injury

AKI is a serious medical condition, characterized by a rapid decline in kidney function, which can lead to progressive renal injury and failure. AKI is associated with high morbidity and mortality, particularly in critically ill patients [42]. It affects a substantial number of individuals with an estimated annual incidence of over 13.3 million cases.

Tragically, AKI contributes to approximately 1.7 million deaths annually. The impact of AKI on public health underscores the urgent need for prevention, early detection, and effective management strategies. AKI is clinically characterized by a sudden and acute decline in renal function, with systemic or localized ischemia and exposure to nephrotoxic substances being the most commonly identified etiological factors. These underlying causes can arise from a multitude of factors such as severe infections, hypovolemia, certain medications, kidney obstruction, and other comorbidities. Early identification and management of causative factors are imperative for the effective diagnosis and the treatment of AKI, given its high incidence and associated morbidity and mortality rates [65]. Although AKI has traditionally been viewed as a self-limiting and reversible condition, it is now recognized that inadequate or maladaptive repair of the tubular epithelium can lead to the progression of kidney injury [66,67]. Over the past few decades, extensive research has been conducted to investigate the pathogenesis of AKI, resulting in the identification and elucidation of several key mechanisms, including inflammation, redistribution of renal blood flow, disturbed cellular bioenergetics, and cell cycle arrest [68]. The hypothesis being investigated is whether USP36 plays a role in the pathogenesis of AKI [40]. This hypothesis proposes an investigation into the involvement of USP36 in AKI, potentially leading to the identification of novel mechanisms and also therapeutic targets. If confirmed, these findings could significantly advance our understanding of AKI and contribute to the development of effective treatment strategies. First, ischemia-induced kidney injury was accompanied by a significant decrease in USP36 levels both in vivo and in vitro. Second, in vitro experiments showed that USP36 overexpression resulted in a significant reduction in tubular epithelial cell apoptosis induced by serum deprivation.

These findings have provided evidence that USP36 contributes to the pathogenesis of ischemia-induced AKI, and suggest that it is a promising therapeutic target for this condition. Further investigation is warranted to elucidate the underlying mechanisms of USP36 involvement in AKI and to more thoroughly evaluate its potential as a therapeutic target [42].

### 5.2. Non-Alcoholic Steatohepatitis

Nonalcoholic fatty liver disease (NAFLD) poses a substantial risk of developing hepatocellular carcinoma (HCC) [69]. In the absence of effective therapeutic interventions, NAFLD may progress to nonalcoholic steatohepatitis (NASH), fibrosis/cirrhosis, and HCC. ALR or hepatic stimulatory substances (HSS) was initially discovered in the regenerating livers of weanling rats [70]. Several previous studies have indicated that ALR confers hepatic protection against diverse hepatotoxic agents such as carbon tetrachloride, D-galactosamine, ethanol, and hydrogen peroxide. The absence of ALR not only compromises the ultrastructural integrity of mitochondria but also impairs their functionality. Specifically, in the livers of ALR-CKO mice, the mitochondrial respiratory capacity is compromised, leading to impaired ATP synthesis. MDM2, an oncoprotein that functions as a crucial repressor of p53 [71], facilitates the ubiquitination and subsequent degradation of ALR, whereas the deubiquitinating enzyme USP36 counteracts this process by removing ubiquitin and preventing ALR degradation. These findings strongly indicate that ALR deficiency resulting from ubiquitination contributes to the progression of steatotic liver to a malignant stage. Co-regulation of ALR by MDM2 and USP36 is vital for maintaining protein stability and appears to play a critical role in governing mitochondrial dynamics. Disruption of mitochondrial dynamics promotes the transition from NASH to HCC in the liver [59].

In summary, this study provided evidence that the regulation of ALR by MDM2 and USP36 through ubiquitination is impeded in the context of NASH-associated HCC induced by WD/CCl4. Diminished ALR expression accelerates the progression of NASH to HCC by disrupting mitochondrial dynamics. Consequently, preserving ALR at an optimal level is crucial for preventing the malignant transformation of steatotic hepatocytes.

### 5.3. Human Papillomavirus

The integration of human papillomavirus (HPV) into the host genome is a prominent characteristic of invasive cervical cancer (ICC) [72]. This study aimed to conduct a comprehensive analysis of the Cancer Genome Atlas (TCGA) dataset, which includes a substantial amount of existing data, such as whole-genome sequencing (WGS), RNA sequencing (RNA-seq), DNA methylation profiles, and updated clinical outcome information. The primary goal was to identify novel genes that play a significant role in the development of cervical cancer. These genes are referred to as integration-detected genes (IDGs) because their investigation originated from their proximity to HPV integration events. Identification and characterization of IDGs may provide new insights into the molecular mechanisms underlying cervical cancer development and progression. Multiple studies have provided evidence that USP36 promotes the proliferation and migration of tumor cells [64]. However, the research revealed a significant correlation between elevated USP36 expression and hypomethylation of the CpG site proximate to the HPV integration site. This specific methylation potentially inactivates the USP36 gene, thereby influencing cellular processes such as cell cycle regulation and apoptosis. Consequently, this can facilitate the onset and progression of cervical cancer. Thus, it is suggested that USP36 may play a pivotal role in both HPV infection and cervical carcinogenesis [64].

### 5.4. Cardiomyopathy

The association between USP36 and cardiomyopathy is predominantly manifested in its function in doxorubicin (Dox)-induced cardiomyopathy (DIC). The expression of USP36 in neonatal rat cardiomyocytes and H9C2 cells subsequent to Dox exposure. Upon silencing USP36, both oxidative stress damage induced by Dox and in vitro apoptosis can be significantly mitigated [63].

## 6. Role of USP36 in Cancer

**Figure 4:**
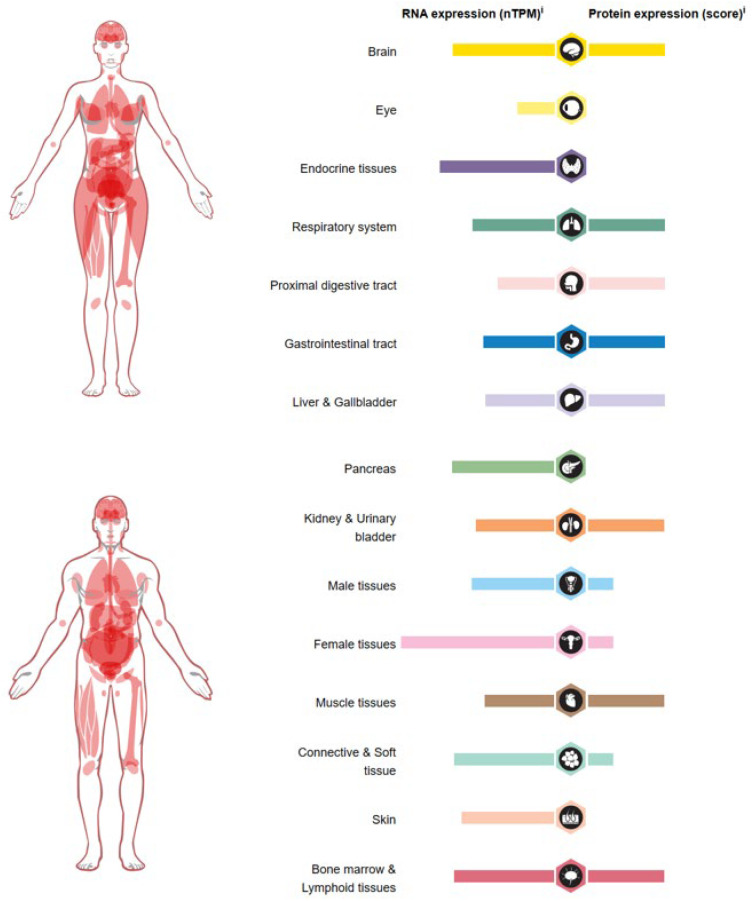
The expression of USP36 RNA and proteins in various human tissues. Available online: https://www.proteinatlas.org/ENSG00000055483-USP36/tissue#rna_expression (accessed on 25 February 2024).

**Figure 5:**
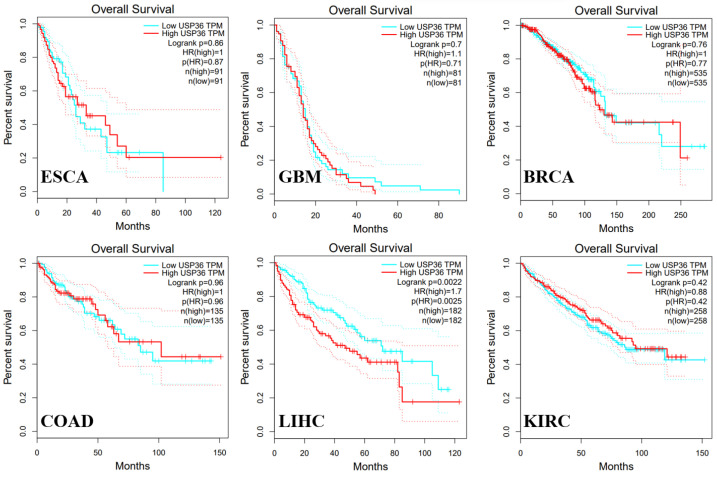
Analysis of the correlation between USP36 expression level and overall survival using Kaplan–Meier test and log-rank test. Esophageal carcinoma (ESCA), glioblastoma multiforme (GBM), breast invasive carcinoma (BRCA), colon adenocarcinoma (COAD), liver hepatocellular carcinoma (LIHC), kidney renal clear cell carcinoma (KIRC). Available online: GEPIA (http://gepia.cancer-pku.cn/, 25 February 2024).

**Figure 6:**
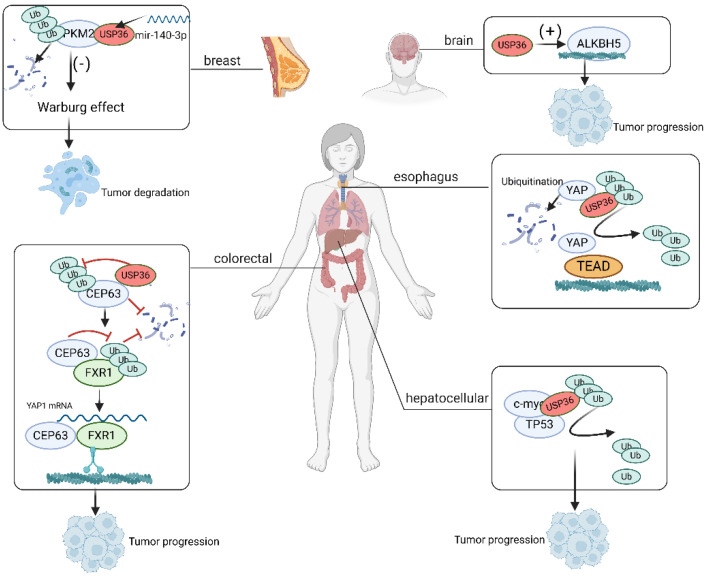
The regulating mechanisms of USP36 in various cancers. This figure was produced by BioRender. (Agreement number: ET26L9E69R).

In humans, USP36 was found to be highly expressed in the brain, endocrine tissues, liver, gallbladder, and female tissues (Figure 4). USP36 is upregulated in multiple cancers including esophageal carcinoma [31], glioblastoma [37], HCC [73], colorectal cancer [74], breast cancer [75], and T cell lymphoma [76]. Although the analysis of Kaplan–Meier survival rate and log-rank test revealed a correlation between USP36 levels and overall survival (OS) in liver hepatocellular carcinoma (LIHC) (Figure 5), there are lots of studies verifying that USP36 is implicated in the progression and drug resistance of many other cancers. The summarized information regarding the function, expression, and targets of USP36 in various cancers (Figure 6 and Table 1) provides a comprehensive resource for understanding the role of USP36 in different pathological conditions. This data compilation will facilitate further research and development of targeted therapeutic systems focused on USP36.

### 6.1. Esophageal Carcinoma

ESCC is a significant global malignancy [78]. The incidence of ESCC exceeds 4,500,000 new cases globally, with approximately half of these cases diagnosed in China [79]. Recent genome-wide sequencing data of ESCC samples has provided evidence suggesting a correlation between Hippo signaling pathway dysregulation and ESCC development [80,81]. Extensive genomic abnormalities, such as mutations and amplifications, have been observed in the Hippo pathway in ESCC. Specifically, mutations in the inhibitory modulators of Hippo signaling, such as AJUBA and FATs, have been identified in ESCC. Although some studies have reported varying requirements for YAP or TAZ in different contexts, YAP/TAZ gene amplification has been consistently detected in ESCC samples [81,82]. Multiple molecular biologically based studies have provided compelling evidence that the depletion of YAP and TAZ can effectively impede the growth of ESCC [83]. Timely detection and implementation of suitable treatment modalities play crucial roles in enhancing the outcomes of patients diagnosed with ESCC [78]. Recent genome-wide sequencing studies of ESCC samples revealed a significant correlation between aberrant Hippo signaling and ESCC. The Hippo pathway is a conserved signaling cascade that governs fundamental cellular events including cell growth, proliferation, and apoptosis. Perturbations in components of the Hippo pathway, such as genetic mutations or dysregulated expression, have been identified in ESCC, suggesting their potential contribution to the pathogenesis of this disease. Exploring the intricate interplay between Hippo signaling and ESCC holds promise for advancing our understanding of the molecular mechanisms underlying ESCC development. However, further comprehensive investigations are necessary to elucidate the precise roles and mechanistic intricacies governing the involvement of Hippo signaling in ESCC [80,81].

### 6.2. Glioblastoma

High-grade gliomas, including glioblastoma multiforme (GBM), are prevalent and highly malignant primary tumors that occur primarily within the intracranial region in adults [84,85]. Despite surgical resection, postoperative adjuvant chemotherapy, and radiation therapy, the prognosis of patients diagnosed with glioblastoma multiforme (GBM) remains unfavorable. The median overall survival (OS) rate following diagnosis typically ranges from 12 to 15 months, with a 5-year survival rate of less than 9.8% [86,87].

ALKBH5 is a well-known m6A demethylase, also referred to as a m6A eraser, that has attracted significant attention from researchers because of its influential role in various biological processes. Abnormal ALKBH5 expression has been implicated in the pathogenesis of various cancers, including glioblastoma [88]. A recent study uncovered a mechanism involving the post-translational modification of the m6A demethylase ALKBH5. USP36 directly cleaves the polyubiquitin chains of ALKBH5, increasing protein stability. Conversely, when USP36 was removed, there was a decrease in ALKBH5 expression, which resulted in the inhibition of tumor growth in glioblastoma xenograft models. Furthermore, a study observed that high USP36 expression is associated with poor prognosis in patients with glioblastoma. There is a direct correlation between elevated USP36 expression and decreased survival in patients diagnosed with glioblastoma. These findings provide valuable insights into the complex molecular interactions involved in tumor formation and highlight the potential of targeting the USP36-ALKBH5 axis as a therapeutic intervention for glioblastoma.

In summary, the studies presented in this context revealed the involvement of PRL1 in facilitating the invasion and migration of GBM through the activation of USP36-mediated Snail2 deubiquitination. These findings shed light on the underlying mechanisms contributing to GBM pathogenesis and provide potential targets for therapeutic interventions. The newly discovered PRL1/USP36/Snail2 axis has emerged as a critical mechanism underlying GBM progression, emphasizing its potential as a therapeutic target [37]. These findings offer insight into novel strategies for developing treatments aimed at disrupting this axis and mitigating GBM progression.

### 6.3. Hepatocellular Carcinoma

Primary liver cancer (PLC) is characterized by increased morbidity and mortality rates, with substantial public health implications. Based on the latest cancer statistics reports, it is estimated that approximately 905, 677 new cases of PLC are diagnosed annually, leading to approximately 830,180 deaths associated with this disease. HCC is the predominant primary liver cancer subtype encountered in clinical practice. During the initial stages of HCC, curative treatment options such as tumor resection (surgical removal of the tumor), ablation (utilizing techniques like radiofrequency or microwave ablation to destroy the tumor), and liver transplantation can be employed as effective therapeutic approaches for managing this disease [89]. A considerable proportion of HCC patients are diagnosed at an advanced stage of the disease. A late-stage diagnosis often limits the range of effective treatment options. Systemic drug therapy is commonly used as a treatment approach. First-line treatments for advanced HCC are often sorafenib and lenvatinib. These treatments typically offer a moderate extension in regard to overall survival, with an average increase of approximately 3 months. Multiple studies have consistently shown that abnormal expression and activity of ubiquitin-specific proteases (USPs) are prevalent in various types of cancer. These findings highlight the importance of USPs in the development and progression of these malignancies [90]. This analysis revealed that a significant majority of USPs displayed distinct expression patterns in HCC, highlighting the widespread presence of abnormal protein ubiquitination in this specific cancer type. A subsequent investigation evaluated the prognostic significance of these dysregulated USPs in HCC and identified more than ten USPs that demonstrated associations with HCC prognosis. Notably, the analysis emphasized the simultaneous and independent effects of USP36 and USP39 on OS, disease-free survival, disease-specific survival, and progression-free survival in patients with HCC. These findings provide strong evidence supporting the conclusion that elevated expression levels of USP36 and USP39 serve as poor prognostic indicators of HCC [73]. Previous studies have suggested that USP36 affects the ubiquitination of c-Myc, thereby influencing its stability within cells [91]. This discovery led to the hypothesis that USP36 potentially participates in the progression of HCC because of its role in stabilizing the protein levels of c-Myc, a known promoter of HCC development. Further empirical investigation is warranted to substantiate this hypothesis. Additionally, it is worth noting that USP36 levels are significantly elevated in *TP53*-mutant HCC, suggesting a potential correlation between USP36 and the molecular characteristics of HCC. Multiple studies have consistently shown that *TP53* is the most frequently mutated gene in HCC and that its abnormal function is closely linked to unfavorable clinical outcomes [92,93]. Previous studies have also explored the potential interplay present between *TP53* gene mutations and USPs. An illustrative example is the positive feedback loop between USP22 and HIF1α, which promotes glycolysis and stemness in HCC following *TP53* mutation [94]. Additionally, it has been observed that USP10 enhances the proliferation of cancer cells in the presence of *TP53* mutations [95]. In summary, these findings indicate a potential collaboration between USP36 and *TP53* mutations to promote HCC progression. Nevertheless, the precise function of USP36 in *TP53*-mutated HCC requires further investigation. Notably, the expression of USP36 is closely linked to the tumor microenvironment, indicating its possible clinical implications. Subsequent research efforts should therefore prioritize the investigation of oncogenic mechanisms through which USP36 operates in HCC, independently validating the reported observations regarding the involvement of USP36 in this specific type of cancer.

### 6.4. Colorectal Cancer

CRC is a widespread malignancy on a global scale, holding the third position in terms of incidence and the second highest in terms of mortality among all types of cancer [80]. Previous studies have documented that the upregulation of CEP63, a component of the centriole, is associated with unfavorable prognosis in cases of neuroblastoma [96]. Elevated CEP63 expression is observed in CRC and correlates with poor prognosis in affected individuals. Previous studies have provided evidence that CEP63 promotes the proliferation of CRC cells and contributes to tumor growth. Interestingly, USP36 stabilizes CEP63 by inhibiting K48 ubiquitination, similar to its role in stabilizing the oncogene c-Myc in CRC [97].

### 6.5. Breast Cancer

Breast cancer is a prevalent malignant tumor [98] and is characterized by significant reprogramming of energy metabolism, which drives malignant progression and unfavorable prognosis [99,100]. The Warburg effect refers to the predominant shift in ATP production within tumor cells, where there is a transition from typical mitochondrial oxidative phosphorylation of glucose to aerobic glycolysis. This phenomenon is characterized by enhanced glucose uptake and lactate production even in the presence of sufficient oxygen, which provides energy and biosynthetic intermediates that support the accelerated proliferation of cancer cells. The Warburg effect is frequently observed in breast cancer and is believed to play a role in tumor growth and progression [101]. Multiple studies have confirmed the high expression of USP36 in breast cancer tumor tissues and its association with poor patient prognosis. The regulatory role of miR-140-3p in controlling USP36 expression has also been elucidated. This regulation affects the expression of pyruvate kinase M2 (PKM2) by modulating the ubiquitination process mediated by USP36. These changes in PKM2 and subsequent alterations in glycolysis contribute to adverse biological processes in breast cancer cells through the Warburg effect.

### 6.6. T Cell Lymphoma

EZH2 has been identified as being overexpressed in various cancer types, including extranodal natural killer/T cell lymphoma (NKTL) [102], where it plays a well-documented oncogenic role. Its overexpression is linked to tumor initiation and progression, leading to unfavorable clinical outcomes. Additionally, increased EZH2 levels are associated with reduced sensitivity to chemotherapy in diverse cancer types [103,104,105]. MELK, a highly conserved AMPK family of serine/threonine kinases, mediates signals initiated by exogenous stimuli. It is overexpressed in a wide range of cancers and facilitates the phosphorylation of the oncogenic transcription factor FOXM1 [106]. In NKTL, MELK overexpression facilitates the specific phosphorylation of EZH2 at S220 and contributes to the stabilization of EZH2 through the involvement of USP36 [76].

## 7. USP36 as a Target for Cancer Therapy

It should be noted that research on USP36 inhibitors is currently limited, and some research may not have been published yet. The development of specific USP36 inhibitors requires further investigation to determine their efficacy, safety, and potential side effects. Therefore, the application of USP36 as a therapeutic target should be approached with caution until further evidence is available. USP36 has been confirmed to play a pro-cancer role across diverse cancer types, and its knockout has demonstrated a substantial reduction in tumor development and migration. Previous studies have shown that miR-140-3p regulates the expression of its downstream target gene, BCL-2. It modulates BECN1-induced autophagy and tumor metastasis in gastric cancer by regulating BCL-2 [107]. In breast cancer, circ_0008039 demonstrates binding affinity for miR-140-3p, thereby modulating its expression, which leads to the inhibition of tumor cell proliferation, migration, invasion, and glycolysis by modulating the miR-140-3p target gene SKA2 [108]. Several studies have reported high USP36 expression in breast cancer tissues, which is strongly associated with a poor prognosis in patients. Additionally, miR-140-3p has been identified as a regulator of USP36 expression. Through its regulatory role, miR-140-3p affects the protein expression of PKM2 by modulating the ubiquitination of PKM2 mediated by USP36. This regulatory mechanism alters glycolysis and other adverse biological processes in breast cancer cells via the Warburg effect [75]. Therefore, USP36 is a promising drug target.

## 8. Conclusions and Future Perspectives

**Figure 7:**
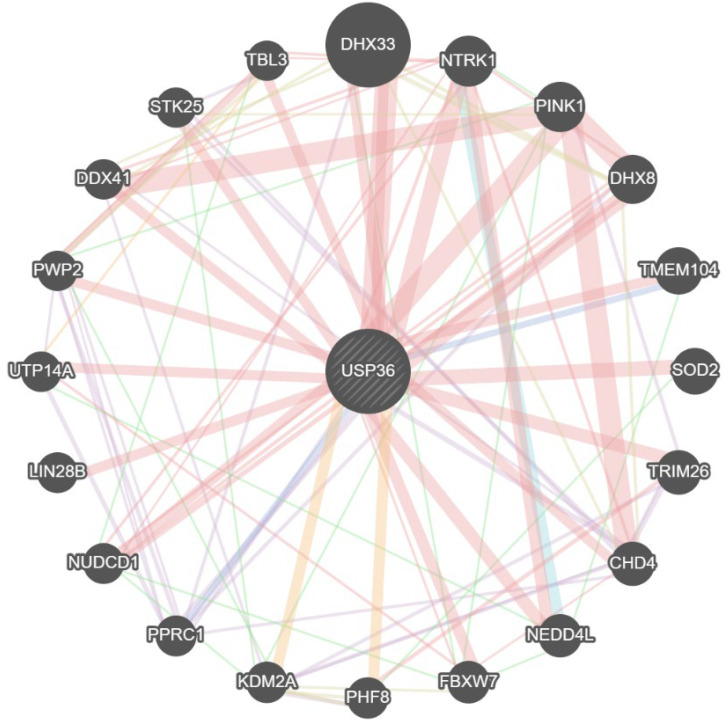
The protein–protein interaction network of USP36 as predicted by the GeneMANA Server.

This review has comprehensively summarized recent studies that have focused on the role of USP36 in cancer and other diseases, highlighting its multifaceted involvement. Relatively few studies have been conducted on USP36. These findings demonstrate that USP36 expression is elevated and plays a significant role in the pathogenesis of various cancers and diseases. Multiple lines of evidence have indicated that USP36 promotes cell proliferation in a wide range of cancers through deubiquitination and stabilization of oncoproteins. Notable examples include breast cancer [75], esophageal carcinoma [31], glioblastoma invasion [37,76], hepatocellular carcinoma [73], colorectal cancer [74], among others. These findings highlight the potential of USP36 as a therapeutic target for treating these diseases. It is important to acknowledge the complexity of USP36’s role in tumourigenesis and recognize that its function may vary depending on the specific context and type of cancer. Consequently, alternative deubiquitinases, such as USP36, have emerged as potential targets for tumor therapy. Further investigation is needed to fully elucidate the intricate mechanisms underlying the dual roles of USP36 in different cancers and to determine the therapeutic potential of targeting USP36 in tumor treatment. MiR-140-3p functions as a promising target for cancer therapy by modulating the expression of USP36, thereby inhibiting tumorigenesis. Additionally, genetic knockout-mediated suppression of USP36 expression has also been observed to effectively impede cancer progression. An effective approach to enhance the selectivity of USP36 antagonists involves designing novel compounds that selectively target the open ubiquitin-binding pocket. By focusing on this specific region, it is feasible to develop inhibitors that can selectively diminish USP36 enzyme activity, thereby minimizing the probability of unintended target interactions. This strategic framework is expected to significantly enhance the selectivity of USP36 inhibitors. Furthermore, the modulation of protein–protein interactions (PPIs) presents another promising approach for augmenting the efficacy and selectivity of targeted therapies [109,110,111]. Through strategic targeting and manipulation of specific PPIs, these interactions can be disrupted or enhanced, resulting in more precise and efficacious therapeutic interventions. By specifically targeting PPIs in the context of USP36 inhibition, potential opportunities arise to interfere with the association of PPIs with other proteins implicated in diverse facets of tumorigenesis and progression. This approach holds promise for enhancing treatment outcomes [110,112]. Like other members of the USP family, USP36 participates in PPIs and exerts biological activity (Figure 7). In conclusion, USP36 inhibitors are promising potential anticancer drugs. Therefore, further development of more potent and selective USP36 inhibitors is warranted. Additionally, the overexpression of USP36 in many tumors contributes to drug resistance, and the discovery of new and effective USP36 inhibitors could be a feasible strategy for overcoming these treatment difficulties in cancer. Furthermore, analyzing the specific biological functions of USP36 and the indication profile of USP36 inhibitors in various cancers will advance the precise treatment of USP36-driven cancers.

## Figures and Tables

**Table 1:** The function of USP36 in varieties of cancers.

References	Functions	Substrates	Cancer Type
[31]	Facilitating ESCC progression via the Hippo/YAP axis	Hippo/YAP	Esophageal carcinoma
[77]	Deubiquitinating Snail2 and thus promoting glioblastoma invasion and tumorigenesis	Snail2	Glioblastoma invasion
[73]	Synergizing with TP53 and promoting HCC progression	TP53	Hepatocellular carcinoma
[74]	Stabilizing CEP63 by reducing its K48 ubiquitination and promoting colorectal cancer progression	CEP63	Colorectal cancer
[75]	Deubiquitinating PKM2 to suppress the malignant phenotypes through the Warburg effect	PKM2	Breast cancer
[76]	USP36 helps mediate the stabilization of EZH2	MELK	T cell lymphoma

## Data Availability

Data is contained within the article.
